# RELATIONSHIPS BETWEEN DEMENTIA CAREGIVER PERSONALITY, OUTCOMES, AND MANAGEMENT AND COPING STRATEGIES

**DOI:** 10.1093/geroni/igad104.2225

**Published:** 2023-12-21

**Authors:** Darby Simon, Amanda Leggett, Emilee Ertle, Benjamin Mast

**Affiliations:** University of Louisville, Louisville, Kentucky, United States; Wayne State University, Detroit, Michigan, United States; University of Louisville, Louisville, Kentucky, United States; University of Louisville, Louisville, Kentucky, United States

## Abstract

Family caregivers of people living with dementia are at higher risk for negative outcomes such as depression and burden. However, the experiences of family caregivers are widely variable due to individual differences. Five-factor model personality traits have previously been connected to depression and burden. The current study seeks to expand this area by investigating the links between personality, caregiver management and coping, and outcomes (burden and depression). The sample consisted of 100 dementia family caregivers ranging from 20-90 years old (X̅=63.7, SD=16.1) who participated in the STYLE study on caregiving. Sample demographics consisted of 76% females, 80% White, 12% Black, 6% other, and 2% declined to report. Multiple regression models revealed significant relationships while controlling for caregiver age, gender, hours of care provided per week, months of providing care, and relationship to care receiver, though burden did not reveal any significant relationships. Higher conscientiousness (β=.226, p=.014) and higher neuroticism (β=.446, p<.001) were associated with higher depression. Higher agreeableness (β=-.367, p<.001) was associated with lower use of criticism-based management strategies, while higher neuroticism (β=.211, p=.023) was associated with higher use of criticism-based management strategies. High openness (β=.230, p=.020) was associated with higher problem-focused coping. Higher agreeableness (β=.201, p=.048) was associated with higher acceptance-focused coping, while higher neuroticism (β=-.254, p=.013) was associated with lower use of acceptance-focused coping. Higher neuroticism (β=.247, p=.020) was associated with higher emotion-focused coping. These analyses demonstrate links between dementia family caregiver personality, the management and coping strategies they use, and caregiver mental health.

